# Fabrication of High Quality, Large Wet Lay-Up/Vacuum Bag Laminates by Sliding a Magnetic Tool

**DOI:** 10.3390/polym10090992

**Published:** 2018-09-05

**Authors:** Marli Sussmann, Mehrad Amirkhosravi, Maya Pishvar, M. Cengiz Altan

**Affiliations:** School of Aerospace and Mechanical Engineering, University of Oklahoma, Norman, OK 73019, USA; marli.m.sussmann-1@ou.edu (M.S.); mehrad@ou.edu (M.A.); pishvar@ou.edu (M.P.)

**Keywords:** polymer-matrix composites, consolidation, lifting magnet, wet lay-up/vacuum bagging, mechanical properties

## Abstract

This study presents a novel method to fabricate high-quality, large composite parts which can be used in a wet lay-up/vacuum bag (WLVB) process. The new method utilizes a commercial lifting magnet, which is commonly used for transporting ferrous plates, to apply a magnetic consolidation pressure on the WLVB composite lay-up. The pressure is applied on a large area of the laminate by slowly sliding the magnet over the vacuum bag surface, which leads to an improved laminate quality. When further improvement is desirable, multiple passes of the magnet can be performed, where each pass successively compacts the lay-up. To explore the feasibility of implementing this technique, random mat and plain weave glass/epoxy laminates were fabricated, and their properties compared to conventional WLVB laminates. The effects of the number of moving passes of the lifting magnet on the laminate microstructure and properties are also investigated. As a result of multiple passes, the fiber volume fraction in random mat and plain weave laminates increases to 34% and 53%, representing 80% and 16% improvements, respectively. In addition, the void volume fraction reduces almost by 60% to a very low level of 0.7% and 1.1%, respectively. Consequently, the flexural properties considerably enhance by 20–81%, which demonstrates the potential of the proposed method to produce WLVB parts with substantially higher quality. It is also shown that there exists an optimal number of passes, depending on the fabric type where additional passes induce new voids as a result of excessive resin removal.

## 1. Introduction

Composite materials are extensively used in structural applications ranging from aerospace [1,2], marine [3,4], and automotive [5,6] applications due to their low weight and high mechanical properties. Among the various composite manufacturing techniques, wet lay-up (aka hand lay-up) vacuum bagging is a relatively low-cost, flexible method that does not require special tooling [7,8]. In this technique, each layer of fiber reinforcement is wetted by liquid resin on a single-sided mold. Then, a squeegee and a roller are used to fully impregnate the fabric and remove entrapped air. This process is repeated for multiple plies until the desired laminate thickness is achieved, and then the vacuum is applied to remove excess resin and volatiles generated during cure. This technique is widely used in fabrication of large, complex composite parts like wind turbine blades [9], marine hulls [10], bridge decks [11], and housing components with relative ease and low cost. In addition, a wet lay-up/vacuum bag (WLVB) is commonly used for structural repair and maintenance of large composite parts [12,13,14,15], whereby on-site restoration of a damaged part is possible without complex tooling.

Despite the advantages and widespread use of WLVB processes for the manufacture and maintenance of medium to large parts, controlling the void content, achieving high fiber volume fraction, and uniformly distributing the resin throughout the laminate are difficult [7]. As a result, the parts made by WLVB have relatively low fiber volume fraction and undesirably high void content [16]. Furthermore, the structural performance and load carrying capability of a part repaired by the WLVB method are often significantly lower than that of the original undamaged part, primarily due to absence of a sufficient consolidation pressure during cure. As an alternative to traditional WLVB process, vacuum infusion processes like vacuum-assisted resin transfer molding (VARTM) are generally used, allowing better control of the fiber-to-resin ratio and marginally reducing the entrapment of air pockets between the plies [17,18]. For comparison, chopped strand, random mat laminates produced by VARTM showed almost 32% higher flexural strength than those made by wet lay-up due to 47% higher fiber volume fraction [19]. However, the consolidation pressure in both VARTM and WLVB processes is limited to the atmospheric pressure, 0.1 MPa, which results in the lower fiber volume fractions and higher void content (e.g., up to 7.5% [20,21,22]) compared to those made under higher pressures of 0.4–1.2 MPa [23,24,25]. For this purpose, high external pressure of more than 0.1 MPa during cure of a composite laminate is required to remove additional resin and suppress or eliminate voids, thus enhancing the mechanical properties. Generally, the high pressure levels can be applied using an autoclave [26,27] or hot press [28,29] both of which require high capital investment and energy consumption. Therefore, the composite industry has been frequently faced with a trade-off between the quality of composite parts and production cost [30].

Accordingly, a cost-effective, flexible method to apply sufficiently high consolidation pressure in WLVB processes can have certain benefits in the manufacture, repair, and maintenance of large composite parts. Towards this goal, Neodymium Iron Boron (NdFeB) permanent magnets have been recently used to generate up to 0.8 MPa compaction pressure on the vacuum bag lay-up [31,32,33,34]. It was shown that by the stationary placement of permanent magnets during cure of the glass/epoxy laminates, both the flexural strength and modulus increased in excess of 46% due to a 70% reduction in void content to less than 2% and a 55% increase in fiber volume fraction. However, covering the entire vacuum bag surface of the medium to large parts by stationary magnets may not be feasible in industrial applications. In addition, handling, placement, and removing a set of permanent magnets may not be easy to perform due to the high levels of magnetic force generated. For instance, 635 kN force can be generated by a set of 2.54 × 2.54 × 1.27 cm^3^, N52 grade, NdFeB magnets covering approximately 1 m^2^ area. Thus, to overcome these concerns, using a hand-held magnetic tool that can be easily slid on a vacuum bag to apply pressure over large areas may be a promising solution. In addition, with such a tool, sliding the magnet multiple times over the same area would be possible, which may lead to a higher level of compaction, and thus, improved laminate quality.

In this paper, we propose for the first time the use of a commercial, hand-held lifting magnet to fabricate high-quality, medium to large WLVB laminates that may be adopted in industrial-scale applications. This device is a light-weight, low-cost tool and can apply a sufficient magnetic compaction pressure on the vacuum bag via the rotation of a simple ON/OFF lever. To generate the magnetic attraction force, the composite lay-up is prepared on a magnetic tool plate. Then, with the use of a suitable lubricant, the magnet is easily slid by hand on the vacuum bag, compacting the entire laminate surface—which is much larger than the area under the magnet. To fully understand the effect of sliding a lifting magnet on the quality of WLVB laminates, two different glass fabrics, chopped strand random mat and plain weave, are selected. Furthermore, the effects of multiple passes of sliding the magnet on the quality of final part are investigated. Finally, the microstructure, void and fiber volume fractions, and flexural properties of the improved laminates are compared to those of laminates fabricated by conventional WLVB without compaction.

## 2. Materials and Methods

### 2.1. Lifting Magnet

A permanent lifting magnet, Mag-Mate PowerLift Magnet model PNL0250 (Industrial Magnetics, Inc., Boyne City, MI, USA) was used for applying external pressure on the composite lay-up, prepared on a magnetic tool plate. Table 1 shows the properties typically reported for the lifting magnet utilized in this study. This tool is lightweight, inexpensive, and is commercially used for holding, carrying, and releasing ferromagnetic metals up to 113 kg (250 lbs).

**Figure 1:**
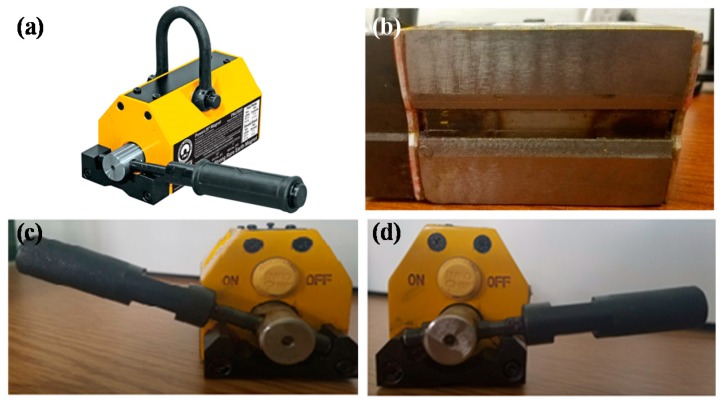

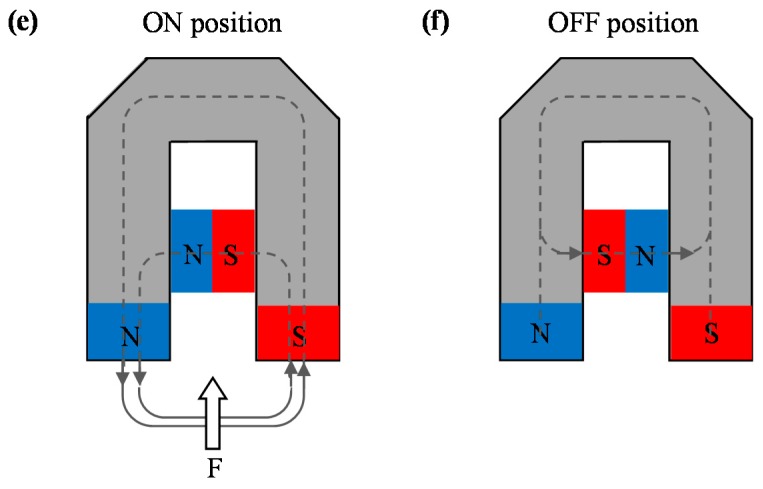
(**a**) General view of lifting magnet; (**b**) bottom surface of the lifting magnet; (**c**) the handle on the lifting magnet at ON position; (**d**) the handle on the lifting magnet at OFF position; (**e**) schematic of magnetic field at ON position; and (**f**) schematic of magnetic field at OFF position.

Figure 1 shows a general view of the lifting magnet (Figure 1a), details on the bottom surface (Figure 1b), the handle on the magnet featuring a lock-on and lock-off mechanism (Figure 1c,d), as well as the magnetic circuit (Figure 1e,f). The lifting magnet is made using two Neodymium magnets, one of which is a stationary horseshoe magnet and the other is a square magnet that can be rotated with the handle. As can be seen in Figure 1e,f, the rotation of the square magnet changes the magnetic circuit which dictates whether the magnetic pressure is ON or OFF. Figure 1c,e show that at the ON position, the magnets are lined up, North to North and South to South, and the magnetic field attracts any ferromagnetic materials placed underneath. By rotating the handle to the OFF position, one field is reversed, and the magnetic field stays inside the magnet, so the magnet does not attract any material (see Figure 1d,f). Thus, only at the ON position, the attraction force is generated between the magnet and the tool plate, which compacts the lay-up in between. This compaction pressure depends on the gap (i.e., lay-up thickness) between the magnet and the tool plate, where the maximum compaction pressure of 731 kPa (106 psi) can be reached at the zero-gap distance, according to the data sheet.

**Figure 2:**
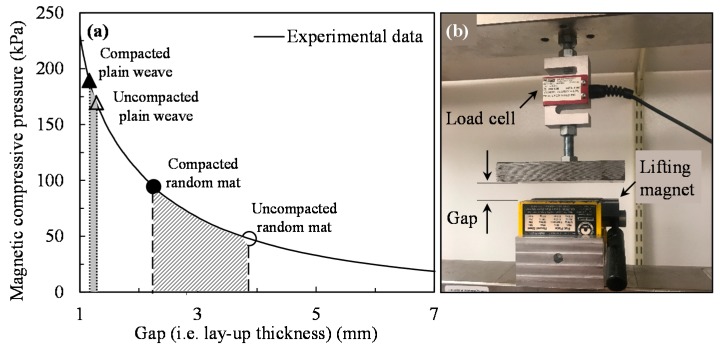
(**a**) Compaction pressure of the lifting magnet versus gap (i.e., lay-up thickness) and (**b**) a photograph of the experimental set-up used for measuring the magnetic pressure. The open and solid circles represent the thickness of uncompacted and compacted 4-ply random mat lay-up. The open and solid triangles correspond to the thickness of uncompacted and compacted 6-ply plain weave lay-up.

Figure 2a illustrates the compaction pressure generated by the lifting magnet versus the gap (i.e., lay-up thickness). A photograph of the experimental set-up used for measuring the magnetic compaction pressure is shown in Figure 2b. First, the magnetic attraction force is measured while varying the gap between the lift surface and the tool plate using a mechanical testing machine. Then, the pressure is obtained dividing the force by the surface area of the magnet. Figure 2a shows that the pressure decreases exponentially with an increase in lay-up thickness. Also, the thickness of a 4-ply random mat lay-up reduces from 3.87 to 2.25 mm during cure due to magnetic compaction pressure, as shown by open and solid circles in Figure 2a. As a result, the magnetic pressure applied on random mat laminates increases from 48 to 94 kPa (6.9–13.6 psi) during cure, as shown in Figure 2a. Similarly, the lay-up thickness consisting of 6-plies of plain weave fabrics decreases from 1.28 to 1.16 mm during cure under magnetic pressure (see open and solid triangles in Figure 2a). Thus, the plain weave laminates are subjected to a higher range of compaction pressure from 170 to 189 kPa (24.7–27.4 psi) due to lower lay-up thickness of the 6-ply plain weave than that of 4-ply random mat.

### 2.2. Composite Constituents

Two different types of reinforcements were chosen in this study. One is E-glass, chopped strand, random mat (Fiber Glast Developments Corp., part#250, Brookville, OH, USA) with an areal density of 0.458 kg/m^2^, and the other is plain weave glass fabric (Hexcel Corp., HexForce 3733, Stamford, CT, USA) with an areal density of 0.197 kg/m^2^. INF-114 epoxy resin and curing agent INF-211 were used as the resin system (hardener to resin ratio was 27.4/100 by weight). This resin has a relatively low viscosity (<100 mPa s at 60 °C), so the applied external pressure will likely cause considerable resin outflow. The resin has a long gel time of approximately 50 min at 60 °C that allowed sufficient time to complete the compaction without a discernible increase in viscosity.

### 2.3. Laminate Manufacturing

Herein, for the first time, a commercial lifting magnet was utilized to apply the compaction pressure on the vacuum bag lay-up during cure. First, the lay-up was prepared on a 6.45 mm-thick magnetic tool plate following the conventional wet lay-up/vacuum bag (WLVB) procedure, and then the lifting magnet was placed on the vacuum bag. Since the area under the magnet is smaller than the surface area of the laminate, the magnet was slid by hand along the length of the laminate to compact the entire surface. It is expected that sliding the magnet while applying compaction pressure on the lay-up would help to squeeze out the excess resin and remove mobile voids, both of which will significantly enhance the mechanical properties of composites. However, the improvements that can be achieved under compaction pressure are influenced by the reinforcement’s type. Basically, the random mat is thicker than the woven fabric and has a greater number of pores in its structure, so it is compacted more under pressure, resulting in higher improvement. In addition, as mentioned in previous studies [35,36,37], applying successive compaction cycles on the preform reorganizes the fiber network and may reduce the resin-rich areas and void content of the final part. Thus, sliding the magnet was performed in one pass, as well as in multiple passes over the entire laminate surface.

In this study, the laminates are fabricated under nine different scenarios listed in Table 2. Two identical laminates are fabricated for each scenario. These scenarios were selected to: (i) assess the feasibility of using a hand-held lifting magnet in WLVB processes; (ii) compare the quality of the random mat and plain weave WLVB laminates made by a lifting magnet with the ones fabricated by conventional WLVB laminates; and (iii) determine the effect of multiple passes of the magnet on the quality of WLVB laminates. In this regard, the baseline laminates, WLVB-RM-4-0 and WLVB-PW-6-0, were manufactured using a conventional WLVB method without applying external pressure. In the WLVB-RM-4-0 laminates, 4 plies of random mat, and in the WLVB-PW-6-0, 6 plies of plain weave fabric, were utilized. Then, for comparison, the random mat and plain weave laminates, WLVB-RM-4-1 and WLVB-PW-6-1, respectively, were fabricated using only one pass of the lifting magnet. Considering that additional resin is removed with each pass of lifting magnet, the number of passes is considered an important parameter affecting the quality of the final part. Thus, WLVB-RM-4-6, WLVB-RM-4-12, and WLVB-RM-4-18 laminates are fabricated by 6, 12, and 18 passes of the magnet on the saturated random mat preforms, respectively. However, for plain weave laminates, WLVB-PW-6-6 and WLVB-PW-6-12, only 6 and 12 passes of the magnet were utilized, respectively. The reason for this is that the lay-up consisting of 6 plies of plain weave is much thinner than the 4 plies of the random mat, so the applied magnetic pressure is much higher. Thus, for plain weave laminates, it is expected that fewer passes would be sufficient to capture the full benefits of magnetic compaction.

In this study, all the laminates consist of either 4 plies of random mat or 6 plies of plain weave, 152.4 mm × 203.2 mm (6″ × 8″) E-glass fabrics. For the matrix material, epoxy and hardener were mixed for 5 min at 350 rpm using a mechanical mixer. The mixture was then degassed around 15 min to remove entrapped air until all visible bubbles disappear. The visual demonstration of the composite lay-up preparation using the WLVB process can be found in Ref. [33]. Briefly, the steps are as follows: the 38.1 × 25.4 cm^2^ area of 400-series stainless steel tool plate was covered by a release film for easy removal of the laminate. Prior to laying the fabric, a coat of resin was applied on the tool plate and spread using a squeegee. The first ply was then placed on the resin, and a stainless-steel roller was used to enhance the impregnation and remove entrapped air. Once this is completed, more resin was poured onto the ply and uniformly spread over the fabric with a squeegee. This process was repeated until all the plies were placed. A 0.3 mm-thick, 152 mm × 203 mm, aluminum caul plate, taped to a piece of 216 mm × 267 mm perforated release film, was sprayed with PTFE release agent and placed on top of the saturated preform. The perforated release film was then taped down to the base plate to create a constraint on resin flow during vacuum and cure. For the random mat laminates, this constraint was 20 mm, and for the plain weave laminates, it was 25 mm away, circumscribing the entire lay-up. Bleeder material is placed on top of the release film to absorb the excess resin, and a thru-bag vacuum outlet connector is then placed on top of the bleeder, away from the preform. A vacuum bag was placed over the entire area and the edges of the bag were sealed to the tool plate. After 45 min from the start of fabric lay-up, the vacuum pump pulled a negative pressure of 95 kPa, and the tool plate was heated to 60 °C by the flexible silicone heat sheets secured to the bottom surface of the tool plate. Both vacuum and temperature were held constant for 8 h until the laminate was fully cured.

### 2.4. Applying the Lifting Magnet during Fabrication

**Figure 3:**
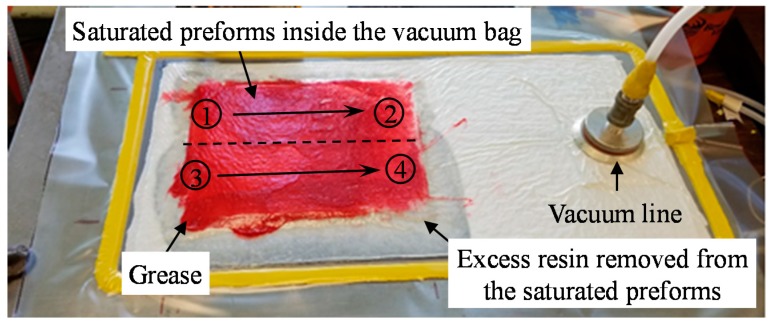
Wet layup/vacuum bag lay-up under vacuum. The magnet was applied to the greased (red) area. A single pass of magnet included compacting the lay-up from point 1 to 2 and point 3 to 4.

To fabricate WLVB laminates using a lifting magnet, the following procedures were employed: after the vacuum was drawn in the bag, anti-seize multi-purpose EP grease (Lucas Oil Products Inc., Corona, CA, USA) was applied to the surface of the vacuum bag to reduce the friction so that the magnet can be manipulated and slid with relative ease. The lifting magnet was wrapped in a vacuum bag material because the friction between the two vacuum bags is much lower than the friction between the aluminum bottom surface of the lifting magnet and the vacuum bag over the laminate. Prior to wrapping the magnet in the vacuum bag, a piece of foam was also placed under the leading edge of the magnet to keep the sharp metal edge from scrapping the grease off the vacuum bag ahead of the magnet. The foam is thin enough (≈0.1 mm) so that there is effectively no increase in the gap between the magnet and the steel tool plate. Figure 3 shows the prepared lay-up under the vacuum, where magnetic pressure is applied to the greased (red) area. The contact area of the lifting magnet was 3.8 cm-wide × 9.2 cm-long which was smaller than the surface area of the saturated preform (15.2 cm-wide × 20.3 cm-long). Therefore, the magnet was slid by hand along two parallel lines from point 1 to 2 and point 3 to 4 (see Figure 3) to cover the entire surface of the vacuum bag. Once the mold temperature reached 60 °C, the magnet with the handle in the OFF position was placed on a corner of the saturated preform (point 1) such that the width of the magnet was along the length of the laminate. The application of the pressure started by rotating the handle to the ON position and then slowly sliding the magnet along the length of the laminate. Once the magnet reached point 2, the handle was switched back to the OFF position and the magnet was lifted off the surface and placed at point 3, so that the next slide covered the entire area of the laminate. Thus, a single pass of magnet compacts the lay-up from point 1 to 2 and point 3 to 4, which took approximately 20 s. The application of magnetic compaction either ended here or was repeated 5, 11, or 17 more times to fabricate laminates with either 1, 6, 12, or 18 total passes. When the number of passes increases, re-application of the grease becomes necessary, so a foam scraper is used to redistribute the grease over the lay-up surface before doing a new pass.

### 2.5. Void and Fiber Volume Fraction Measurement

Void and fiber volume fraction of composite laminates fabricated under different scenarios are determined according to ASTM D3171-15 by conducting resin burn-off tests on the samples as per ASTM D2584-11. For this purpose, six 38 × 19 mm^2^ specimens were characterized from each scenario. The density of samples was determined using the suspension method, where distilled water is added to a heavy liquid solution from Cargille Laboratories that has a density of 2.49 g/cm^3^ at 23 °C until the sample no longer floats and is suspended in the solution. Then, the specimens were placed in a 600 °C oven for 4 h to determine the weight fraction of composite constituents.

Two thermogravimetric analysis (TGA, Q50, TA Instruments, New Castle, DE, USA) tests were also conducted for the fabrics, and the results show that at 600 °C, a 2.46% and 0.21% mass loss occurred in the random mat and plain weave fabrics, respectively. This fiber mass loss, which probably occurred due to the burning off of the fiber sizing, is considered in the determination of the fiber volume fraction. The density of the glass fibers taken from the random mat and plain weave fabrics were also obtained with a nitrogen pycnometer, and were 2.603 ± 0.004 and 2.600 ± 0.003 g/cm^3^, respectively. The density of cured neat resin, obtained by the suspension method, was 1.152 ± 0.003 g/cm^3^.

### 2.6. Scanning Electron Microscopy: Sample Preparation and Imaging

A scanning electron microscope (SEM, Zeiss Neon 40 EsB model, Carl Zeiss AG, Oberkochen, Germany) was used to characterize the void morphology, content, and location within the composite laminate [38,39,40]. It is also used to visually inspect the thickness and nominal fiber volume fraction of the final part. Two 25.4 mm-long specimens were cut from each laminate, mounted in an acrylic resin, thoroughly polished, and sputter coated by gold/palladium. Then, SEM images of the specimens were taken using a Zeiss Neon 40 EsB model. Magnifications of 35× and 150× were used to capture images of the voids, fibers, and resin that make up the plain weave laminates, and magnifications of 20× and 150× were used to view the microstructure of the random mat laminates. The location and shape of larger voids, as well as the compaction of the fiber plies, can be seen from the lower magnification SEM images. The higher magnifications were used to characterize smaller voids located in and along the individual fiber tows.

### 2.7. Characterization of Mechanical Properties

The mechanical properties of random mat and plain weave laminates made with conventional WLVB and increasing number of passes of the lifting magnet were characterized with the flexure test. The three-point bending method was used to determine the flexural strength and modulus of the materials. This test is performed using a Com-Ten^®^ 705TN testing machine (Com-Ten Industries Inc., Pinellas Park, FL, USA) at a cross-head speed of 2 mm/min. Random mat and plain weave specimens were tested at span-to-thickness ratios of 20 and 24, respectively. The flexural stress (*σ_f_*) applied during this test was determined using,

(1) $$\;\sigma_{f}=\frac{3FL}{2bd^{2}}\;,\;$$

where *F* is the force measured by the load cell, *L* is the length of the support span, and *b* and *d* are the width and thickness of the composite sample, respectively. The elastic modulus (*E_f_*) can also be determined using,

(2) $$\;E_{f}=\frac{L^{3}m}{4bd^{3}}\;,\;$$

where *m* is the slope of the load-deflection curve. However, in cases where the deflection (*D*) is greater than 10% of the span, the flexural stress becomes

(3) $$\;\sigma_{f,large\;deflection}=\sigma_{f}\left[1+6{\left(\frac{D}{L}\right)}^{2}-4\left(\frac{d}{L}\right)\left(\frac{D}{L}\right)\right]\;,\;$$

where a correction factor is added to account for the large deflection of the sample in accordance to ASTM D790-17.

## 3. Results and Discussion

### 3.1. Laminate Thickness, Fiber Volume Fraction, and Void Content

#### 3.1.1. Random Mat

**Figure 4:**
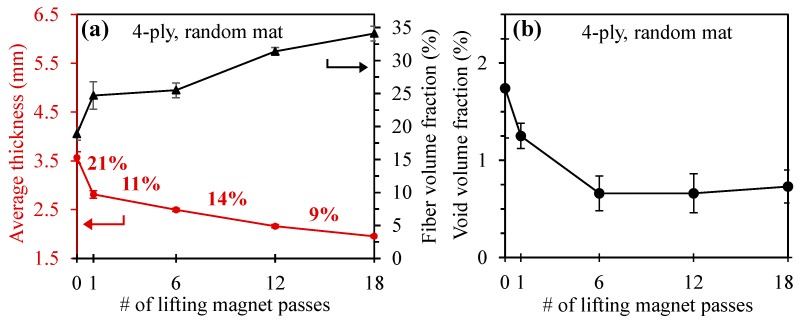
(**a**) Thickness, fiber volume fraction, and (**b**) void volume fraction of random mat laminates fabricated with wet lay-up/vacuum bag (WLVB) and with an increasing number of magnet passes (i.e., 0, 1, 6, 12, and 18 passes). Note: Error bars show the 95% confidence interval (*n* = 6 samples for fiber volume fraction and void volume fraction; *n* = 70 samples for laminate thickness). The percentages shown in (**a**) correspond to the percent thickness reduction between number of passes labeled in the figure.

The reduction in the laminate thickness and improvements of the fiber volume fraction and void content of 4-ply, random mat laminates made by sliding the magnet multiple times (i.e., 1, 6, 12, and 18) are presented in Figure 4a,b. The numerical values of these laminate properties are also summarized in Table 3, where n refers to the number of samples tested.

The average thickness of random mat laminates fabricated using conventional WLVB (WLVB-RM-4-0) was measured to be 3.57 ± 0.12 mm, which was substantially reduced to 1.95 ± 0.03 mm after 18 passes of the magnet (WLVB-RM-4-18), as depicted in Figure 4a. Figure 4a also shows that the 95% confidence interval of the 0-pass laminate thickness has a relatively high value of ±0.12 mm. This high variation and nonuniformity in the thickness of 0-pass laminates is due to spatial variation of planar density of random fiber mats and the presence of resin-rich regions. However, as shown in Figure 4a, the variation of laminate thickness can be significantly reduced from ±0.12 to ±0.03 mm as the consolidation pressure is applied by sliding the magnet multiple times towards the exit gate over the vacuum bag. The significant effect of applying external pressure on reducing the variation of the laminate thickness was also reported in Ref. [41].

The highest percent decrease in laminate thickness is approximately 21% after the first pass of the magnet. The subsequent passes only lead to 9–14% additional thickness reduction, resulting in a 45% total thickness reduction after 18 passes. Thus, the first pass of the lifting magnet caused the highest amount of resin out-flow, probably due to the high permeability of the random mat and presence of resin-rich regions between the plies. As the number of magnet passes increases, the saturated preforms become more compact, and the permeability of the fabric decreases, and thus, the amount of resin out-flow reduces.

As can be seen in Figure 4a, the reduction in the laminate thickness correlates with the increase in the fiber volume fraction, where the fiber volume fraction of the laminate improved by 80% from 19% to 34% due to consolidation pressure applied by 18 passes of the magnet.

Figure 4b illustrates that the void content of the 0-pass laminate is 1.74% and is reduced to 0.66% by either 6 or 12 passes of the magnet, which shows a 62% total reduction in the void volume fraction. Although the void volume fraction decreases with 6 and 12 passes of the magnet, after 18 passes the void volume fraction slightly increased to 0.73%. The reason is that by increasing the number of passes from 12 to 18, excessive resin is squeezed out, and dry spots start to form, thus inducing new voids into the laminate. The increase in the void content can be the limiting factor in deciding the maximum number of passes that should be applied without having any adverse effects due to magnetic compaction.

#### 3.1.2. Plain Weave

**Figure 5:**
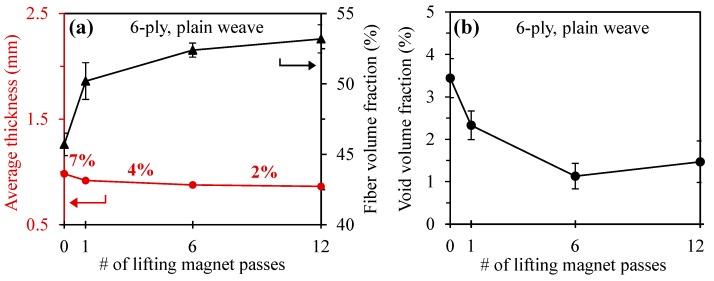
(**a**) Thickness, fiber volume fraction, and (**b**) void volume fraction of plain weave laminates fabricated with WLVB and increasing number of magnet passes (i.e., 0, 1, 6, and 12 passes). Note: Error bars show the 95% confidence interval (*n* = 6 samples for fiber volume fraction and void volume fraction; *n* = 70 samples for laminate thickness). The percentages shown in (**a**) correspond to the percent thickness reduction between number of passes labeled in the figure.

Figure 5a illustrates that the decrease in thickness of 6-ply plain weave laminates follows a similar trend as the random mat laminates (see also Table 3 for comparison of the numerical values). The thickness of plain weave laminates decreased by 7, 11, and 12 percent after 1, 6, and 12 passes of the magnet, respectively. As can be seen in Figure 5a, the thickness curve at 12 passes almost reaches an asymptote. The smaller percentage decrease in the thickness of plain weave laminates compared to the random mat ones can be explained by Darcy’s law, where the resin out-flow rate is directly proportional to the permeability of the fabric. Basically, random mat fabrics have higher permeability than plain weave fabrics [42] because of the random orientation of the fibers and their higher porosity. Therefore, due to the lower permeability of plain weave fabrics, the rate of resin out-flow in plain weave is lower. Figure 5a also shows that unlike random mat laminates, variation in the thickness of plain weave laminates made by conventional VARTM is very low (i.e., 95% confidence interval of ±0.01). This low level of variation in the thickness of the laminates is due to the uniform, structured orientation of fibers in the woven fabric [14].

As can be seen in Figure 5a, the fiber volume fraction, like the thickness of the plain weave laminates, quickly approaches an asymptote. The maximum fiber volume fraction is 53.1% after 12 passes, which shows a 16.2% improvement compared to the 0-pass laminates. Thus, compared to random mat laminates, which show an 80.4% increase in fiber content, the 16.2% improvement achieved in plain weave laminates is much lower. The reason is that the baseline fiber content of plain weave (46%) is much higher than that (19%) of the random mat laminates.

The void volume fraction of plain weave laminates after 6 passes of magnet (WLVB-PW-6-6) reduces by 67.2 to 1.13%, as given in Figure 5b and Table 3. Similar to the random mat laminates, increasing the number of passes on the plain weave lay-up after a certain point (i.e., 12 passes) results in an increase in the void content from 1.13% to 1.47%, due to squeezing out too much resin and creating dry spots. Therefore, in plain weave laminates the increase in void content limits the maximum number of passes to 6, even though the fiber volume fraction continues to show a slight improvement.

Overall, regardless of the fabric type used, the sliding magnet tool proved to be capable of reducing final thickness, increasing fiber volume fraction, reducing void content, and improving thickness uniformity of the composite parts. In addition, sliding the magnet multiple times over the entire laminate enables further compaction of the preform and leads to an improved laminate quality. However, there exists an optimal number of magnet passes, after which the void content increases. This optimal number depends on the fabric type, which is found to be 12 passes for random mat and 6 passes for plain weave.

### 3.2. Microstructural Analysis

#### 3.2.1. Random Mat

**Figure 6:**
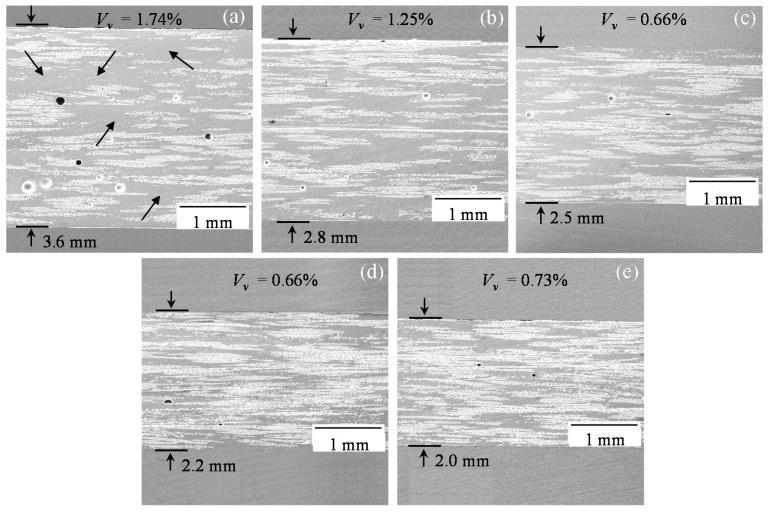
Scanning electron microscopy (SEM) images of random mat laminates (at 20× magnification) made by WLVB process and sliding lifting magnet over the saturated lay-up with (**a**) 0 pass (arrows show the resin-rich inter-tow regions); (**b**) 1 pass; (**c**) 6 passes; (**d**) 12 passes; and (**e**) 18 passes.

Figure 6 provides a side-by-side comparison of cross-sectional SEM images captured at 20× magnification from the random mat samples made by the WLVB and increasing number of magnet passes (i.e., 0, 1, 6, 12, and 18). Figure 6 clearly shows that only one pass of magnet uniformly reduces the laminate thickness, and a higher number of passes results in additional thickness reduction. In 0-pass laminates, large resin-rich areas are formed, as shown by arrows in Figure 6a, due to the low compaction pressure in the WLVB process. The resin-rich areas create weak points in the part, leading to reduced mechanical properties [43]. Figure 6b,e clearly present reduced resin-rich regions as well as improved compaction of preforms by sliding the magnet multiple times. The reason is that by increasing the number of passes, the laminate thickness is reduced, which leads to an exponential increase in magnetic compaction pressure, and thus, more resin is removed. In addition, it was reported that applying successive compaction cycles reorganizes the fiber network and slightly moves the fibers into empty spaces, leading to reduced resin-rich areas [35].

The 0-pass random mat laminates (Figure 6a) contain a high frequency of large, spherical voids which correlate to their high void volume fraction of 1.74%. These voids are mostly observed in the resin-rich regions of the material. The presence of large voids is expected to be detrimental to the mechanical properties due to their deleterious effects on the crack propagation in the resin-rich, inter-tow regions [44,45,46,47]. Voids are also one of the concerns for long-term durability of composites as they accelerate moisture uptake [48]. Figure 6b illustrates that with one pass of the magnet in WLVB-RM-4-1 laminates, the number of voids as well as their size are reduced, both of which decrease the void content compared to that in conventional WLVB laminates (WLVB-RM-4-0). Two factors contribute to the reduction in voids. One is the removal of mobile voids with resin out-flow and the other is suppression of voids between plies under applied pressure, as discussed in the literature [49]. After 6 or more passes, voids become less frequent, smaller, and even slightly elongated which agrees with the void volume fractions of 0.66–0.73% obtained for the 6 to 18-pass laminates.

#### 3.2.2. Plain Weave

**Figure 7:**
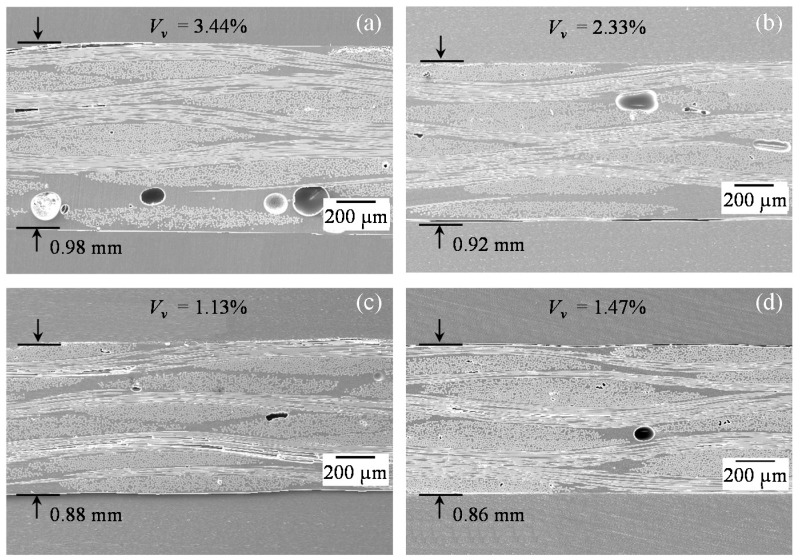
Scanning electron microscopy (SEM) images of plain weave laminates (at 35× magnification) made by WLVB process and sliding lifting magnet on the saturated lay-up with (**a**) 0 pass; (**b**) 1 pass; (**c**) 6 passes; and (**d**) 12 passes.

Figure 7 shows through-the-thickness cross-sectional SEM images of plain weave laminates made by WLVB and increasing number of magnet passes (i.e., 0, 1, 6, and 12) at 35× magnification. The reduction in the thickness of plain weave laminates caused by increasing the number of lifting magnet passes can be clearly observed in SEM images (Figure 7), which confirms the results reported in Figure 5. Unlike random mat, it is possible to distinguish the individual plies in plain weave laminates, which allows clear observation of improved ply consolidation. In the unpressurized case (see Figure 7a), thicker resin-rich inter-tow regions are observed in the laminate, especially between the lower plies. Figure 7b demonstrates that a single pass of magnet removes excess resin from inter-tows regions and improves the consolidation of the laminate. By 6 and 12 passes, inter-tow gaps continue to narrow, as can be seen in Figure 7c,d. Also, comparing Figure 7a–d, it is clear that with increasing number of passes, fiber volume fraction is improved, which corroborates the burn-off results of a 16.2% increase in fiber content from the 0-pass case to the 12-pass case, as given in Figure 5.

The frequency and morphology of voids in the plain weave laminates made under different scenarios can also be seen in the SEM images of Figure 7. Since the fibers are tightly aligned, full wet out of tows are more difficult than that in the random mat, thus in addition to a number of large voids between the tows, a few small voids are seen inside the tows (see Figure 7). The presence of these large and small voids substantiates the high void content of 3.4% in conventional WLVB laminates (WLVB-PW-6-0). With increasing number of passes of magnet from 0 to 1 and 6, large voids between the tows become smaller, which decreases the overall void content (see Figure 7b–d).

**Figure 8:**
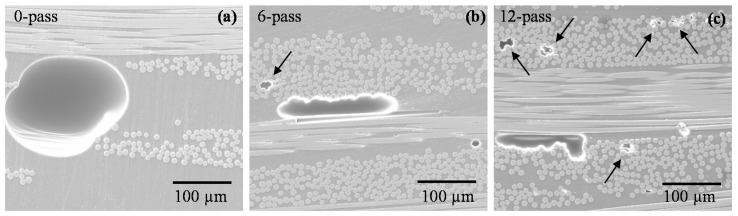
Scanning electron microscopy (SEM) images of plain weave laminates (at 150× magnification) made by WLVB process and sliding lifting magnet on the saturated lay-up with (**a**) 0 pass; (**b**) 6 passes; and (**c**) 12 passes. Note: Arrows point to the small voids inside the fiber tows.

The increased void content in the laminates made by 12 passes compared to that in 6 passes can be explained by comparing their high-magnification SEM images. Figure 8 shows the representative SEM images of plain weave laminates (at 150× magnification) made with 0, 6, and 12 passes of the magnet. Figure 8a illustrates a large void trapped in the resin-rich inter-tow region of the laminate made by conventional WLVB. As the external pressure is applied by sliding the magnet, large, spherical voids become elongated between the fiber tows, as can be seen in Figure 8b,c. The second type of voids, shown by arrows in Figure 8b,c, are the smaller voids inside the fiber tows. These small voids are formed as the number of passes increases from 6 to 12 due to squeezing out too much resin and creating dry spots (see Figure 7 and Figure 8), thus leading to an increase in void content. Applying external pressure may decrease voids between the tows by suppressing or removing them but does not reduce the small voids inside the tows, because of the difficulty in removing this type of void [15].

### 3.3. Flexural Strength and Modulus

#### 3.3.1. Random Mat

**Figure 9:**
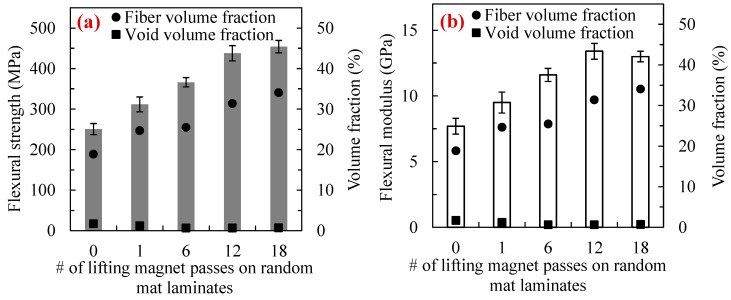
(**a**) Flexural strength and (**b**) modulus of random mat laminates fabricated with WLVB and increasing number of passes of a lifting magnet (i.e., 0, 1, 6, 12, and 18 passes). Void and fiber volume fraction values are also presented. Note: Error bars show the 95% confidence interval (*n* = 14 samples).

Figure 9a,b show the flexural strength and modulus of the random mat laminates made with WLVB process and sliding lifting magnet over the lay-up with 0, 1, 6, 12, and 18 passes. The fiber and void volume fraction values are also given to discern their effects on flexural properties of the laminates.

Figure 9 shows that by increasing the number of magnet passes from 0 to 12, both the flexural strength and modulus of laminates improve significantly. This confirms that strength and modulus are fiber-dominant properties where an increase in fiber volume fraction directly affects the mechanical properties of the laminates. The flexural strength increases by 24% from 250.7 MPa in conventional WLVB laminates to 311.7 MPa with only one pass of the magnet. Similarly, the flexural modulus increases by 23% from 7.7 to 9.5 GPa. The subsequent passes (6 and 12) lead to a 17–40% additional enhancement in flexural properties, resulting in a 74% total increase in both flexural strength and modulus after 12 passes. After 18 passes, flexural strength of laminates increases only 4% while flexural modulus decreases by 3% compared to 12 passes, probably due to having a higher void content with a similar fiber volume fraction. Therefore, by sliding the magnet 12 times on the random mat lay-up one can achieve the highest improvement in overall quality of the parts. The results also substantiate that the proposed method can cause a remarkable improvement in quality of large composite parts even by a single pass of the magnet. Sliding the magnet with one pass also gives the flexibility of using fast curing resin systems and thus reduces the total fabrication time and labor cost which are of interests in composite manufacturing.

#### 3.3.2. Plain Weave

**Figure 10:**
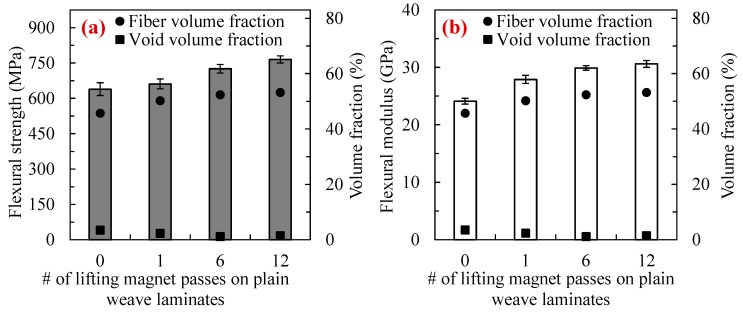
(**a**) Flexural strength and (**b**) modulus of plain weave laminates fabricated with WLVB and increasing number of passes of a lifting magnet (i.e., 0, 1, 6, and 12 passes). Void and fiber volume fraction values are also presented. Note: Error bars show the 95% confidence interval (*n* = 14 samples).

Figure 10a,b show the flexural strength and modulus as well as void and fiber volume fractions of the plain weave laminates made with using the WLVB process and sliding the magnet over the lay-up with 0, 1, 6, and 12 passes.

Since the plain weave fibers are tightly arranged, and the baseline fiber volume fraction of these laminates made by conventional WLVB is high (i.e., *V_f_* ≈ 46%), their flexural properties do not increase as much as that of random mat laminates by applying external pressure. It is evident that as the number of passes increases, the flexural strength and modulus of laminates gradually increases by 3–20% (from 638.9 to 765.2 MPa) and 16–27% (from 24.1 to 30.6 GPa), respectively. The considerable improvements observed in flexural properties can mainly be ascribed to the increase in fiber content by 10–16% and reduction of voids by 32–67%. According to the results, the laminates made with 6 passes showed 14% and 24% improvements in flexural strength and modulus, respectively, as well as the lowest void content (i.e., *V_v_* = 1.13%). The laminates made with 12 passes demonstrated the highest flexural strength and modulus, but a slightly higher void content than 6-pass laminates. The flexural properties of the plain weave laminates almost reach their asymptote with 12 passes of the lifting magnet. With that in mind, and the findings regarding increases in void content, passes over 12 are not recommended for plain weave laminates.

## 4. Conclusions

This study introduced a new technique that can be used for improving the quality of large composite parts made by the wet lay-up/vacuum bag (WLVB) process. For this purpose, a magnetic consolidation pressure was applied by sliding a hand-held lifting magnet over a large area of the vacuum bag lay-up. Using a suitable lubricant on the vacuum bag reduced the friction between the magnet and the vacuum bag lay-up and helped to easily slide the magnet with a single pass as well as multiple passes. The effectiveness of this inexpensive and portable tool in improving the quality of WLVB laminates was demonstrated by fabricating random mat and plain weave laminates.

It was shown that by using multiple passes of the magnet, the fiber volume fraction of random mat laminates was enhanced from 18.9% to 34.1%, indicating an 80% improvement, while the void content was decreased from 1.74% to 0.73%. As a result, their flexural strength and modulus were improved by 81% to 454.0 MPa and by 74% to 13.4 GPa, respectively. The plain weave laminates had a higher baseline fiber content of 45.7%, but still, sliding the magnet could effectively improve their fiber content by 16% to 53.1% while reducing the voids fraction from 3.44% to 1.13%. Meanwhile, the flexural strength and flexural modulus of plain weave composites with multiple passes of magnet were increased by 20% to 765.2 MPa and by 27% to 30.6 GPa, respectively. It is worth noting that regardless of the type of fabric, the increase in mechanical properties was substantial in the first pass, and in the subsequent passes a considerable improvement was observed. However, comparison of microstructural observations and void content revealed that an optimal number of passes exists depending on the fabric type, where additional passes induces new voids because of excessive resin removal. Consequently, sliding the magnetic tool presented in this work provides a practical and effective approach for fabricating high-quality WLVB parts, which can be utilized for manufacturing larger composite parts in a variety of industrial applications.

## Figures and Tables

**Table 1:** Properties of lifting magnet, Mag-Mate PowerLift Magnet model PNL0250, used in this study.

Magnetic Characteristic	Values
Magnet material	Neodymium
Working load limit (flat)	113 kg
Weight	3.17 kg
Overall length × width × height	12.7 × 6.7 × 6.7 cm^3^
Contact surface area	35 cm^2^
Maximum operating temperature	82 °C

**Table 2:** Designations for the laminates fabricated with different fabric types and different manufacturing processes.

Fabrication Scenario	Manufacturing Process	Fabric Type	No. of Plies
WLVB-RM-4-0	Conventional WLVB	Random mat	4
WLVB-RM-4-1	WLVB with 1 pass of lifting magnet		
WLVB-RM-4-6	WLVB with 6 passes of lifting magnet		
WLVB-RM-4-12	WLVB with 12 passes of lifting magnet		
WLVB-RM-4-18	WLVB with 18 passes of lifting magnet		
WLVB-PW-6-0	Conventional WLVB	Plain weave	6
WLVB-PW-6-1	WLVB with 1 pass of lifting magnet		
WLVB-PW-6-6	WLVB with 6 passes of lifting magnet		
WLVB-PW-6-12	WLVB with 12 passes of lifting magnet		

**Table 3:** Thickness and fiber and void volume fractions for 4-ply random mat and 6-ply plain weave laminates fabricated with an increasing number of passes (i.e., 1, 6, 12, and 18 passes for random mat and 1, 6, and 12 passes for plain weave) of a lifting magnet (*n* = 6 for fiber volume fraction and void volume fraction; *n* = 70 for thickness of random mat laminate; and *n* = 126 for thickness of plain weave laminate, 95% confidence intervals for all data).

Fabrication Case	Thickness (mm)	Fiber Volume Fraction (%)	Void Volume Fraction (%)
WLVB-RM-4-0	3.567 ± 0.119	18.9 ± 1.0	1.74 ± 0.56
WLVB-RM-4-1	2.811 ± 0.079	24.7 ± 2.4	1.25 ± 0.15
WLVB-RM-4-6	2.496 ± 0.035	25.5 ± 1.2	0.66 ± 0.20
WLVB-RM-4-12	2.158 ± 0.034	31.4 ± 0.7	0.66 ± 0.22
WLVB-RM-4-18	1.954 ± 0.031	34.1 ± 1.2	0.73 ± 0.19
WLVB-PW-6-0	0.982 ± 0.009	45.7 ± 0.8	3.44 ± 0.46
WLVB-PW-6-1	0.918 ± 0.007	50.2 ± 1.5	2.33 ± 0.37
WLVB-PW-6-6	0.877 ± 0.004	52.4 ± 0.6	1.13 ± 0.33
WLVB-PW-6-12	0.863 ± 0.006	53.1 ± 1.1	1.47 ± 0.54
